# Plasma polymerized nanoparticles are a safe platform for direct delivery of growth factor therapy to the injured heart

**DOI:** 10.3389/fbioe.2023.1127996

**Published:** 2023-06-20

**Authors:** Zoë E. Clayton, Miguel Santos, Haisam Shah, Juntang Lu, Siqi Chen, Han Shi, Shaan Kanagalingam, Praveesuda L. Michael, Steven G. Wise, James J. H. Chong

**Affiliations:** ^1^ Westmead Institute for Medical Research, Sydney, NSW, Australia; ^2^ Sydney Medical School, University of Sydney, Sydney, NSW, Australia; ^3^ School of Medical Sciences, Faculty of Health and Medicine, The University of Sydney, Sydney, NSW, Australia; ^4^ Charles Perkins Centre, The University of Sydney, Sydney, NSW, Australia; ^5^ Cardiology Department, Westmead Hospital, Sydney, NSW, Australia

**Keywords:** nanoparticles, platelet derived growth factor (PDGF), cardiomyocytes, myocardial infarction, coronary artery smooth muscle cells, cardiac fibroblast

## Abstract

**Introduction:** Heart failure due to myocardial infarction is a progressive and debilitating condition, affecting millions worldwide. Novel treatment strategies are desperately needed to minimise cardiomyocyte damage after myocardial infarction and to promote repair and regeneration of the injured heart muscle. Plasma polymerized nanoparticles (PPN) are a new class of nanocarriers which allow for a facile, one-step functionalization with molecular cargo.

**Methods:** Here, we conjugated platelet-derived growth factor AB (PDGF-AB) to PPN, engineering a stable nano-formulation, as demonstrated by optimal hydrodynamic parameters, including hydrodynamic size distribution, polydisperse index (PDI) and zeta potential, and further demonstrated safety and bioactivity *in vitro* and *in vivo*. We delivered PPN-PDGF-AB to human cardiac cells and directly to the injured rodent heart.

**Results:** We found no evidence of cytotoxicity after delivery of PPN or PPN-PDGFAB to cardiomyocytes *in vitro,* as determined through viability and mitochondrial membrane potential assays. We then measured contractile amplitude of human stem cell derived cardiomyocytes and found no detrimental effect of PPN on cardiomyocyte contractility. We also confirmed that PDGF-AB remains functional when bound to PPN, with PDGF receptor alpha positive human coronary artery vascular smooth muscle cells and cardiac fibroblasts demonstrating migratory and phenotypic responses to PPN-PDGF-AB in the same manner as to unbound PDGF-AB. In our rodent model of PPN-PDGF-AB treatment after myocardial infarction, we found a modest improvement in cardiac function in PPN-PDGF-AB treated hearts compared to those treated with PPN, although this was not accompanied by changes in infarct scar size, scar composition, or border zone vessel density.

**Discussion:** These results demonstrate safety and feasibility of the PPN platform for delivery of therapeutics directly to the myocardium. Future work will optimize PPN-PDGF-AB formulations for systemic delivery, including effective dosage and timing to enhance efficacy and bioavailability, and ultimately improve the therapeutic benefits of PDGF-AB in the treatment of heart failure cause by myocardial infarction.

## 1 Introduction

Heart failure caused by myocardial infarction is a debilitating condition with high morbidity and mortality. Acute myocardial infarction results in the loss of millions of highly specialised cardiomyocytes, which are unable to regenerate in any meaningful capacity and are instead replaced by non-contractile scar tissue. This leads to permanent and progressive impairment of the pump function of the heart. Despite substantial advances in cardiovascular drug and device therapy, heart transplantation remains the only cure for end-stage heart failure. There is an urgent unmet need for novel treatment strategies to minimise cardiomyocyte death after myocardial infarction and promote repair of the injured heart.

One such strategy is platelet derived growth factor (PDGF) therapy. PDGF is a potent stimulator of cell migration and proliferation, particularly in the context of new blood vessel formation (angiogenesis) and wound healing. There are four PDGF ligands (A, B, C, D), which function as dimers (AA, AB, BB, CC, DD). The heterodimer ligand, PDGF-AB, signals though both PDGF receptors, PDGFRα and PDGFRβ. PDGFRα is present on many cardiac cell types, including cardiac fibroblasts and vascular smooth muscle cells. Recently published research in preclinical models of myocardial infarction has shown that treatment with recombinant PDGF-AB protein (Asli et al., 2019; Thavapalachandran et al., 2020) and overexpression of the *Pdgf-a* gene via recombinant adeno-associated virus mediated gene transfer (Rashid et al., 2021) improves cardiac function after myocardial infarction via fibroblast and macrophage activation and enhanced angiogenesis in the infarct border zone, leading to improved scar alignment and mechanics.

These studies administered PDGF-AB via systemic infusion. However, previous studies have demonstrated that pretreatment with PDGF via direct intramyocardial delivery can also be effective in limiting cardiac damage after infarction (Edelberg et al., 2002; Xaymardan et al., 2004). As PDGF-AB is reported to have a short half-life in the circulation and is susceptible to degradation by proteases, we investigated a nanocarrier-mediated direct delivery method to extend the bioavailability of PDGF-AB during the critical post-infarct period, without the need for continuous systemic infusion (Cianciolo et al., 1999; Barrientos et al., 2008). Carbon-activated, plasma polymerized nanoparticles (PPN) are a high-throughput, low-cost strategy for localized delivery of bioactive cargo (Santos et al., 2018; Santos et al., 2019). PPN can effectively bind and deliver functional siRNAs and pharmaceuticals to target cancer cells (Michael et al., 2020). They have undergone comprehensive cytotoxicity testing in immortalized cell lines, endothelial cells, and smooth muscle cells, but have not been tested in cardiomyocytes nor delivered directly to the heart (Michael et al., 2021).

Here, we show that application of therapeutically relevant doses of PPN and PPN-PDGF-AB to cardiac cells, including primary and pluripotent stem cell derived cardiomyocytes, does not detrimentally affect their viability or function *in vitro*. We also demonstrate that PDGF-AB protein retains its functional efficacy while bound to PPN and establish the potential of PPN as a suitable platform to deliver growth factors and other therapeutic materials to the injured heart.

## 2 Methods

### 2.1 PPN-PDGFAB formulation

#### 2.1.1 PPN synthesis

PPN were produced in a custom-built capacitively coupled radiofrequency (rf) plasma chamber as previously described (Santos et al., 2018). Briefly, an rf-driven (13.52 MHz) plasma discharge was created at a pressure of 110 mTorr, by ionization of a gaseous mixture comprising of argon, nitrogen, and acetylene. The flow rate of each gas was set constant and monitored by mass flow controllers throughout the process. The rf power was delivered by an rf power supply and coupled to the plasma via an automatic matching box to minimize reflected power. The plasma emission fingerprint was monitored *in-situ* via optical emission spectroscopy (OES) using an Ocean Optics HR4000 (Oceans Optics). The OES process is used for quality control purposes, by tracking the emission dynamics of selected molecular species, including molecular nitrogen excited states and ions, as well as CN molecular radicals.

#### 2.1.2 PPN collection

The nanoparticles produced within the plasma bulk were collected in 24-well polystyrene plates (Corning) directly from the active plasma volume as previously described (Santos et al., 2019). Following synthesis, the plates were brought to atmospheric pressure and removed from the vacuum chamber for storage at room temperature and in ambient air for 48 h before resuspension in aqueous solution. Since PPN are produced in a dry plasma state, they can be stored in a dry state and only be resuspended in solution as needed before functionalization with molecular cargo. Here, PPN were dispersed in ultra-pure nuclease-free water (NFW) directly from the well plates, into 1.5 mL Eppendorf tubes. The concentration of PPN in solution was determined by UV/VIS spectroscopy as previously described (Michael et al., 2020), by measuring the optical density of PPN in solution at 500 nm.

#### 2.1.3 PPN characterization

The hydrodynamic parameters of PPN, i.e., hydrodynamic size, polydisperse index (PDI) and zeta potential, were obtained using a Zetasizer Nano ZS (Malvern Instruments, Germany). Samples were measured at room temperature in disposable folded capillary cells (Malvern DTS1070) and data for each hydrodynamic parameter was acquired from the average of five independent acquisitions. The quality of the measurements was monitored in real time and the data was analyzed using standard procedures within the manufacturer’s software (Malvern Instruments, Germany). The hydrodynamic size distribution was further confirmed in a higher resolution NanoSight NS300 laser light scattering system. Samples were introduced into the analysis chamber and allowed to flow at a constant flow rate. The trajectories and Brownian motion of PPNs flowing through the chamber were continuously tracked for 60 s, using a nanoparticle tracking and analysis software (NanoSight NTA 3.0).

#### 2.1.4 PPN-PDGFAB conjugation

Conjugation of PDGF-AB to PPN was performed in ultrapure NFW, by adding PDGF-AB (1 mg/mL) to PPN solutions (10^10^ PPN/mL) at a ratio of 5 µg PDGFAB/10^10^ PPN. The ratio between PDGFAB and PPN during the conjugation process ensures that PDGFAB is present in excess amount compared to what is theoretically necessary to fully decorate the surface of PPNs with a monolayer of PDGFAB molecules, i.e., 3.1 µg/10^10^ PPN. The PPN and PDGF-AB mixtures were incubated for 1 h at 4°C. Unbound PDGF-AB remaining in solution following conjugation was washed by centrifugation at 10,000 G for 5 min.

### 2.2 Cell culture

#### 2.2.1 Neonatal rat ventricular myocytes

All NRVM collection procedures had ethical approval (Western Sydney Local Health District animal ethics protocol 4232) and were performed in accordance with the National Health and Medical Research Council (NHMRC) Code for the care and use of animals for scientific purposes.

NRVMs were obtained from neonatal hearts at postnatal day 3. The hearts were placed in trypsin and incubated at 4°C on a shaking platform overnight. The following morning, the hearts were further dissociated with repeat washes in 1 mg/mL collagenase in HBSS. The cells were collected, centrifuged, resuspended in M199 + 10% fetal bovine serum (FBS), and filtered using a 40 μm cell strainer. The cells were then pre-plated onto T150 flasks for 1 hour, to reduce fibroblast contamination of the NRVM cultures. NRVMs in suspension were then centrifuged, counted, resuspended in fresh M199 + 10% FBS and re-plated onto gelatin-coated 48 well plates for downstream assays.

#### 2.2.2 Human coronary artery vascular smooth muscle cells

Primary HCASMCs were purchased from ATCC (Lot # 61646600). The cells were cultured on T25 and T75 flasks, in Smooth Muscle Cell Growth Medium-2 (SmGm-2, Lonza) at a seeding density of 2500–5,000 cells/cm^2^. Media was replaced every 2–3 days. Replicates were sourced from 3 different donors and used for experiments between passage 3 and 5.

#### 2.2.3 Human cardiac fibroblasts

Human cardiac fibroblasts were isolated from 3 donor heart samples provided by the Sydney Heart Bank. The cells were expanded in complete MEMα culture medium (Thermofisher Scientific) supplemented with 20% FBS, 4 mM L-glutamine, and 100U Penicillin/Streptomycin. The fibroblasts were cultured in T150 flasks and passaged when they reached approximately 80% confluence. Culture media was removed, and the cells were washed twice with PBS. Cells were detached with 2X trypsin‐EDTA for 4 min at 37°C. Trypsin‐EDTA was deactivated by adding double volume complete medium to trypsin-EDTA. The cell suspension was transferred to a 50 mL falcon tube and centrifuged at 250 *g* for 5 min at room temperature to pellet cells and remove trypsin. Cells were resuspended in growth medium for reseeding.

#### 2.2.4 Human pluripotent stem cell derived cardiomyocytes

Human embryonic pluripotent stem cells (HES3 NKX2.5^eGFP/w^) were cultured in Geltrex (Thermofisher)-coated T75 flasks, in TeSR-E8 + Penicillin/Streptomycin (STEM CELL Technologies). For differentiation, ESCs were dissociated at 80% confluency and seeded onto Geltrex-coated 6 well plates at a density of 2.5 × 10^5^ cells per well, in TeSR-E8 + Pen/Strep + 1 µM Y-27632 (Rock inhibitor). Media was replaced with fresh TeSR-E8 + Pen/Strep the following day.

Human induced pluripotent stem cells (SCVI8 - Stanford Cardiovascular Institute) were cultured on Matrigel (Corning) -coated 60 mm dishes, in mTeSR Plus (STEMCELL Technologies) complete media. For differentiation, iPSCs were dissociated at 80%–90% confluency and seeded onto Matrigel-coated 6 well plates at a density of 1.5 × 10^6^ cells per well, in mTeSR Plus +1 µM Y-27632. Media was replaced with fresh mTeSR Plus the following day.

For both cell lines, cardiomyocyte differentiations commenced 2 days after replating, using an established small molecule protocol described by Lian et al. (2013). Briefly, the cells were incubated in RPMI 1640 + B27 minus insulin + Glutamax + Pen/Strep + 6 µM CHIR99021 for 24 h. Media was then changed to fresh RPMI 1640 + B27 minus insulin + Glutamax + Pen/Strep. On day 3 the cells were incubated with 5 µM Wnt signaling inhibitor, IWP-2. Media was changed to fresh RPMI 1640 + B27 minus insulin + Glutamax + Pen/Strep on day 5 and then to RPMI 1640 + B27 (with insulin) + Glutamax + Pen/Strep from day 7 onwards. PSC-CMs commenced beating on day 7–8, with robust spontaneous contractions observed from day 10 onwards.

### 2.3 *In Vitro* assays

#### 2.3.1 DCFDA (2′,7′ –dichlorofluorescin diacetate) reactive oxygen species assay

After 14 days of differentiation, iPSC-CMs (SCVI8) were dissociated from 6 well plates with TryPLE and replated into Matrigel-coated wells of a 24 well plate. The cells incubated overnight in RPMI1640 + B27 + Glutamax + Penicillin/Streptomycin + 1 µM Y-27632 (Rock inhibitor) and media was changed to fresh RPMI1640 + B27 + Glutamax + Penicillin/Streptomycin the following day. Three days after replating, all wells had resumed beating and cells were treated with PPN or PPN-PDGFAB.

DCFDA cellular reactive oxygen species (ROS) assay (ab113851, Abcam) was performed on iPSC-CMs after 24 h incubation with 1 × 10^8^ or 1 × 10^9^ PPN or PPN-PDGFAB, according to the manufacturer’s instructions for pretreatment of adherent cells and with volume modifications for the 24-well plate format. Four hours prior to the end of the incubation period, media was removed from 3 wells and the cells were treated with 55 µM TBHP positive control compound in 400 µL supplemented buffer. One hour before the end of the incubation period, media was removed from all other wells and replaced with 400 µL supplemented buffer containing 20 µM DCFDA. DCFDA (20 µM) was also added to TBHP wells. The plate was incubated in the dark for 45 min, transferred to the microplate reader without washing, and fluorescence was read at 485/535 nm. The experiment included cell-only controls (no PPN/PPN-PDGFAB) and blank wells (no cells).

#### 2.3.2 MTT (3-(4, 5-dimethylthiazol-2-yl)-2, 5-diphenyltetrazolium bromide) viability assay

For viability and mitochondrial activity assays, NRVMs were seeded onto gelatin-coated 48 well plates at a density of 1 × 10^5^ cells/well and incubated for 24 h in. The following day, the cells were washed three times with PBS and incubated with fresh M199 + 10% FBS. On d2, the media was switched to M199 + 2% FBS and cells were incubated with PPN or PPN-PDGFAB at high (1 × 10^8^ particles), medium (5 × 10^7^ particles) or low (2.5 × 10^7^ particles) doses, or PBS only control. MTT and TMRE assays were performed on d1, d4, d7, d14 and d21 post-treatment with PPN/PPN-PDGFAB. Each assay was performed using 3 different NRVM preps (n = 3 technical replicates per biological replicate, n = 3 biological replicates per time-point).

MTT was diluted in RPMI 1640 without phenol red, at a concentration of 5 mg/mL. The solution was filtered and added to the cells at a final concentration of 0.5 mg/mL. The cells were incubated at 37°C for 4 h in the dark and then washed twice with PBS. A solvent (500µL, 1:1 DMSO: Isopropanol) was added to each well to dissolve the formazan crystals. The absorbance of each well was measured using a microplate spectrophotometer at 570 nm. Each experiment included appropriate control and blank wells (solvent only).

#### 2.3.3 Tetramethylrhodamine, ethyl ester mitochondrial membrane activity assay

TMRE mitochondrial membrane potential assays were performed using commercially available reagents (ab113852, Abcam, Cambridge, United Kingdom), per the manufacturer’s instructions. The cells were washed with PBS and incubated in M199 + 2% FBS +500 nM TMRE for 25 min in the dark. The cells were then washed twice with 0.2% BSA in PBS and the fluorescence in each well was measured using a microplate spectrophotometer (Spectramax) at Ex/Em 549/575 nm. Each experiment included cells only (no TMRE) and FCCP inhibitor controls.

#### 2.3.4 Boyden chamber migration assay

Human coronary artery vascular smooth muscle cells were seeded in Corning Transwell polycarbonate membrane cell culture inserts (8 μm pore size) at a density of 1.5 × 10^4^ cells in 100 µL DMEM +10% FBS per insert. The transwells were inserted into 24 well plates containing 600 µL DMEM +10% FBS + either 10 ng/mL rhPDGF-AB, 1 × 10^8^ NP3, or 2.5 × 10^7^, 5 × 10^7^, or 1 × 10^8^ PPN-PDGF-AB. The cells were incubated overnight and on the following morning the membranes were washed with 1 mL PBS and fixed in 650 μL 70% ethanol for 30 min. The membranes were then scraped with a 1 mL syringe tip to remove non-migrated cells and washed with PBS. The membranes were cut away from the transwells using a scalpel blade, mounted on microscope slides with mounting media (Vectashield with DAPI (Vector Laboratories Inc.) to stain cell nuclei), and coverslipped. The membranes were imaged on a fluorescent microscope (Olympus BX50), using the ×10 objective.

#### 2.3.5 Myofibroblast differentiation

Human cardiac fibroblasts were reseeded onto 12 mm glass coverslips laid into 24 well plates once reaching approximately 80% confluence as described above. 2.5 × 10^4^ cells were seeded onto the glass coverslips and cultured in complete MEMα culture medium overnight. Cells were then washed in PBS and cultured in serum starved MEMα culture medium supplemented with 4 mM L-glutamine and 100U Penicillin/Streptomycin. Media was replaced every 24 h with serum starved MEMα culture medium. At 72 h, cells were then treated with one of the following conditions, Serum Starved control, rhPDGF-AB (10 ng/ml), 1 × 10^9^/ml PPN-PDGFAB, 1 × 10^9^/ml PPN only. Media was replaced every 24 h with fresh media and growth factor/PPN for 72 h.

#### 2.3.6 Immunocytochemistry

For myofibroblast differentiation of human cardiac fibroblasts, wells with glass coverslips were aspirated and cells were fixed in 10% neutral buffered formalin for 15 min, permeabilised in 0.1% Triton X-100/PBS for 10 min and blocked for 1 h in 10% normal goat serum (NGS)/0.01% Triton X-100/PBS. Primary antibodies (Table 1) were diluted in 10% NGS/0.01% Triton X-100/PBS and added to coverslips overnight at 4°C. The following morning, the cells were incubated for 1 h with secondary antibodies (Table 1) diluted in 10% NGS/0.01% Triton X-100/PBS, incubated with 1 μg/ml DAPI/PBS for 10 min. Glass cover slips were then mounted in 50% Glycerol/PBS and attached to glass slides.

**TABLE 1:** Antibodies for flow cytometry and immunocytochemistry.

Target	Host	Clone	Vendor	Catalogue #
Anti-PDGFRα-BV786	Mouse	αR1	BD Biosciences	742,669
Anti-vimentin	Rabbit		Cell Signaling	5,741
Anti-smooth muscle alpha actin	Mouse		DAKO	M0851
Anti-sarcomeric alpha actinin	Rabbit		Abcam	68,167

#### 2.3.7 Imaging and analysis

Cells were imaged for immunofluorescence at the Cell Imaging Facility at the Westmead Institute for Medical Research using the VS.120 Slide Scanner with ×20 objective and DAPI, FITC, Texas-Red, and Cy5 channels. 3 × 3mm areas of DAPI positive cells were imaged on each glass slide.

Immunocytochemistry images acquired from the VS. 120 were analysed using FIJI (ImageJ) software. Masks were generated through thresholding images equally. FIJI fill holes and watershed functions were used to acquire full DAPI positive nuclei and the ‘analyze particles’ function removed false negative signals that were not of consistent nuclei size. Masks were then layered using binary reconstruct plugin to acquire αSMA/Vimentin/αSMA + Vimentin only positive cells. Positive cell counts were acquired through the FIJI ‘analyse particles’ function.

#### 2.3.8 Cardiomyocyte contraction analysis using Musclemotion

After 14 days of differentiation, PSC-CMs were dissociated from 6 well plates with TryPLE (Thermofisher) and replated onto Geltrex-coated wells of a 24 well plate. One well of the 6 well plate was replated evenly into 4 wells of the 24 well plate. The cells incubated overnight in RPMI1640 + B27 + Glutamax + Penicillin/Streptomycin + 1 µM Y-27632 (Rock inhibitor) and media was changed to RPMI1640 + B27 + Glutamax + Penicillin/Streptomycin the following day. Experiments commenced when all wells had resumed beating.

The cardiomyocytes were visualised using a light microscope (Olympus), under the ×40 objective. For Musclemotion analysis, 20–40 s video recordings of spontaneous beating were taken on an iPhone 11, using a smartphone digiscoping adapter (Gosky Optics) attached to the microscope eyepiece. Immediately following baseline recordings, 1 × 10⁹ PPN or 1 × 10⁹ PPN-PDGFAB were added directly to the wells. Subsequent 20–40 s video recordings were taken at days 2, 4, and 7. Media changes were performed every third day as normal. All video recordings (.mov file format) were obtained at 60 frames per second and converted to.avi files using open-source software, FFMPEG. Contraction profiles were then generated using the MUSCLEMOTION plugin for ImageJ/FIJI as previously described by Sala et al. (Sala et al., 2018).

### 2.4 *In Vivo*

#### 2.4.1 Left anterior descending artery ligation model of myocardial infarction

Male Sprague-Dawley rats were housed at constant room temperature and humidity, with *ad libitum* access to food and water, and a 12-h light/dark cycle. All animal *in vivo* procedures had ethics approval (Western Sydney Local Health District animal ethics protocol number 5166.10.20) and were performed in accordance with the National Health and Medical Research Council (NHMRC) Code for the care and use of animals for scientific purposes.

Rats aged 10–16 weeks were allocated to one of four treatment groups; Control, PDGF-AB, PPN, or PPN-PDGF-AB prior to experiments commencing. The rats were anaesthetized with isoflurane (5% in O_2_) and ketamine (20 mg/kg, i.p.), intubated, ventilated and maintained on 2% isoflurane for the duration of the procedure. A left anterolateral thoracotomy was performed, and the ribs and lung retracted to expose the heart. The left anterior descending (LAD) coronary artery was ligated with a 5-0 prolene suture. Vessel occlusion was confirmed by blanching of the left ventricular (LV) anterior wall distal to the suture. The researcher performing ligation surgery was blinded to treatment group.

Immediately after ligation, a 31G needle was used for intramyocardial injection of a 50 µL solution containing one of four treatments: saline vehicle only, 100 ng recombinant PDGF-AB, 1 × 10^9^ nanoparticles, or 1 × 10^9^ nanoparticles with bound PDGF-AB. The dose of 1 × 10^9^ PPN-PDGFAB is estimated to be equivalent to 310 ng of recombinant PDGF-AB. Our reasoning for opting for a higher (equivalent) dose of PPN-PDGFAB was to account for the assumption of full surface coverage of the nanoparticles and that as PPN and PPN-PDGFAB readily cross the plasma membrane we anticipated some degree of PPN-PDGFAB internalization by cardiac cells, which may reduce the availability of PDGF-AB to its surface bound receptor. After delivery of injections, the chest wall, overlying muscle, and skin were sutured closed. The rats were treated with antibiotic (enrofloxacin 2.5 mg/kg s.c.) and pain relief (buprenorphine 0.05 mg/kg, s.c.).

#### 2.4.2 Echocardiography

Transthoracic echocardiography was performed at baseline (prior to infarct), 3 days post-infarct and 14 days post-infarct, using a Philips Envisor C ultrasound and pediatric probe. M-mode measurements were taken in the parasternal short-axis view (pSAX - LV Mid). End systolic (LVESD) and end diastolic (LVEDD) diameters were measured for 3 consecutive cardiac cycles per view. Six separate views were recorded by two operators. Fractional shortening measurements reported represent the average of three measurements by one operator at each time-point. Final sample sizes for echocardiography analysis in each treatment group were n = 5 (PPN-PDGFAB), n = 6 (Control), or n = 7 (PPN and PDGFAB) biological replicates. Bland-Altman analysis was performed to measure inter-operator variability of echocardiography measurements (Donner et al., 2018; Bunting et al., 2019).

#### 2.4.3 Histology and immunohistochemistry

Rat hearts were fixed in 10% neutral buffered formalin and then cut into two blocks, apex, and mid-ventricle, using a rodent heart matrix and microtome blades. The tissue was paraffin-embedded and cut into 4 µm sections. Sections were deparaffinized in xylene and hydrated by sequential incubation in 100%, 95%, 70%, 50% ethanol, and water. Sections from both blocks were stained with 0.1% Picrosirius Red +0.1% Fast Green to distinguish collagen infarct area from healthy myocardium. Infarct size was calculated as a percentage of the total LV area.

Adjacent sections from apex and mid-ventricle blocks were used for immunohistochemistry. Antigen retrieval was performed using heated sodium citrate buffer and the sections were washed in PBS +0.1% Tween 20, blocked with 5% goat serum in PBS +0.05% Tween-20, and stained with primary antibodies overnight at 4°C (Table 2). The following day, the sections were washed and incubated with secondary antibodies (Table 2), then washed and incubated with DAPI (1 μg/mL, Sigma-Aldrich/Merck). Coverslips were mounted with PBS: Glycerol.

**TABLE 2:** Antibodies for immunohistochemistry.

*Primary Antibodies*				
Marker	Host	Dilution	Vendor	Product
Alpha smooth muscle actin	Mouse	1:200	Dako	M0851
Cardiac troponin T	Mouse	2.5ug/mL	DSHB	CT3
CD31	Rabbit	1:50	Abcam	ab28364
Collagen type I	Rabbit	1:150	Cell Signalling Technology	91,144
Collagen type III	Rabbit	1:300	Proteintech	22734-1-AP
*Secondary Antibodies*				
Alexa Fluor 488	Goat anti-mouse	1:500	ThermoFisher	A-11029
Alexa Fluor 594	Goat anti-rabbit	1:500	ThermoFisher	A-11012

#### 2.4.4 Imaging and analysis

Immunofluorescence and brightfield microscopy were performed on an Olympus VS. 120 Slide Scanner (Olympus^®^ Corporation, Tokyo, Japan), with the ×10 objective (for brightfield), ×20 objective (UPLSAPO ×20/NA 0.75, WD 0.6/CG Thickness 0.17 or the ×40 objective (UPLSAPO ×40/NA 0.95, WD 0.18/CG Thickness 0.11–0.23) for IHC. Images were acquired using Olympus VS-ASW 2.92 software and processed using Olympus VS-DESKTOP 2.9.

Researchers were blinded to treatment group during image analysis. Images for vessel analysis were prepared by selecting up to 10 fields of view (0.5 × 0.5 mm) from the infarct border zones and infarct scar core regions. Vessel density was measured by counting the number of CD31+/ɑSMA + vessels in each 0.25 mm^2^ field of view and comparing the mean density between samples. All analyses were performed using FIJI software (ImageJ, U. S. National Institutes of Health, Bethesda, Maryland, United States of America). Collagen sub-type analysis was performed using the “color threshold” plugin to measure the area of target staining relative to the total area of the left ventricular myocardium.

### 2.5 Statistics

Data are presented as mean ± standard error of the mean (SEM). Normally distributed assay and histology measurements were analysed by ordinary one-way or two-way ANOVA with Tukey adjustment for multiple comparisons. Non-normally distributed assay and histology measurements were analysed by Kruskal–Wallis test by ranks with Dunn’s adjustment for multiple comparisons. Fractional shortening measurements were analysed by two-way repeated measures ANOVA with Tukey adjustment for multiple comparisons. Echocardiography inter-operator variability (bias) was measured by Bland-Altman analysis. Statistical analyses were performed using GraphPad Prism software (version 9.4.1), with *p* values <0.05 considered statistically significant. Raw data files and full-size image files are available on request.

## 3 Results

### 3.1 Characterisation of PPN-PDGF-AB

**FIGURE 1:**
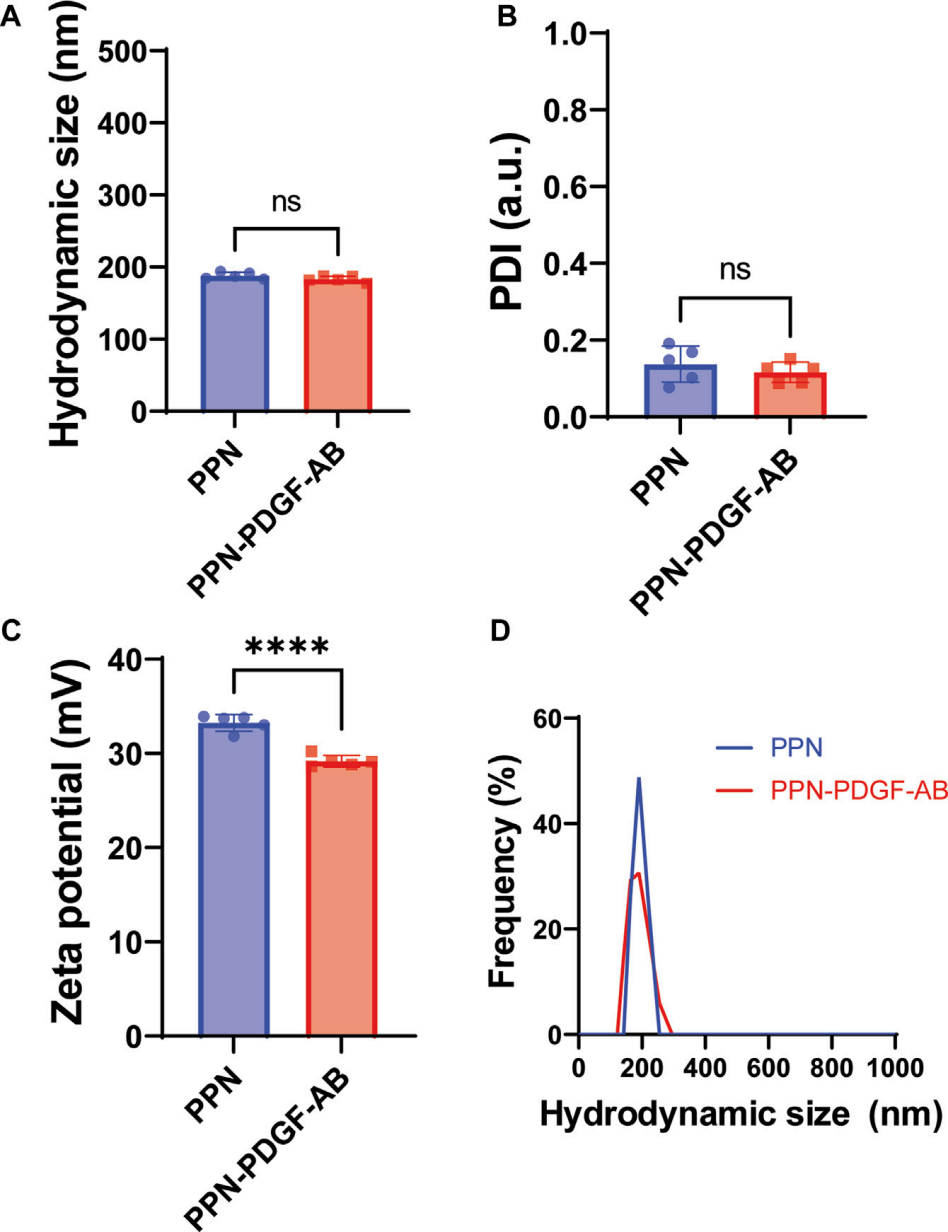
Hydrodynamic parameters of PPN-PDGF-AB formulation. Hydrodynamic size **(A)**, polydisperse index **(B)** and zeta potential **(C)** of pristine PPN and PDGF-AB functionalized PPN. Conjugation of PDGF-AB to PPN yielded stable nano-constructs as indicated by a low PDI and negligible change in hydrodynamic size compared to pristine PPN. The decrease in zeta potential of the PPN-PDGF-AB formulation compared to pristine PPN suggests binding and retention of PDGF-AB to PPN following conjugation. The resulting net surface charge of the nano-constructs allows for stabilization of the formulation via electrostatic repulsion. **(D)**. Hydrodynamic size distribution of PPN and PPN-PDGF-AB formulation in ultra-pure NFW is single-peaked, indicating the formation of monodisperse formulations without aggregation.

We measured the hydrodynamic parameters of PPN, including size distribution, polydisperse index (PDI) and zeta potential in ultrapure nuclease-free water (NFW) using dynamic light scattering (DLS). The process yielded dispersions of pristine PPN with a mean hydrodynamic diameter (Figure 1A), polydisperse index (Figure 1B) and zeta potential (Figure 1C) of 187.9 nm ± 4.6 nm, 0.14 ± 0.05, and 33.2 mV ± 0.9 mV, respectively. Functionalization of PPN with PDGF-AB was performed in NFW by simply mixing PDGF-AB to PPN solutions, following incubation for 1 h at 4°C. The ratio between PDGF-AB molecules to PPN upon conjugation was optimized based on our previous work, which established a relationship between the molecular weight of the molecular cargo and the number of molecules necessary to achieve a full monolayer on the surface of each PPN. Based on the molecular weight of PDGF-AB (26.4 kDa), our model estimates a total of 7.04 × 10^3^ PDGF-AB molecules in a single PPN, or 3.1µg/10^10^ PPN. Functionalization of PPN with PDGF-AB resulted in a stable formulation, as shown in Figure 1D by non-significant changes to median hydrodynamic size and PDI compared to pristine PPN. Measurements showed a decrease in the zeta potential of PPN-PDGF-AB (29.2 mV ± 0.6 mV) relative to unfunctionalized PPN (*p* < 0.001), indicating binding and retention of PDGF-AB on the surface of PPN.

### 3.2 PPN and PPN-PDGFAB is non-toxic to cardiac cells *in vitro*

**FIGURE 2:**
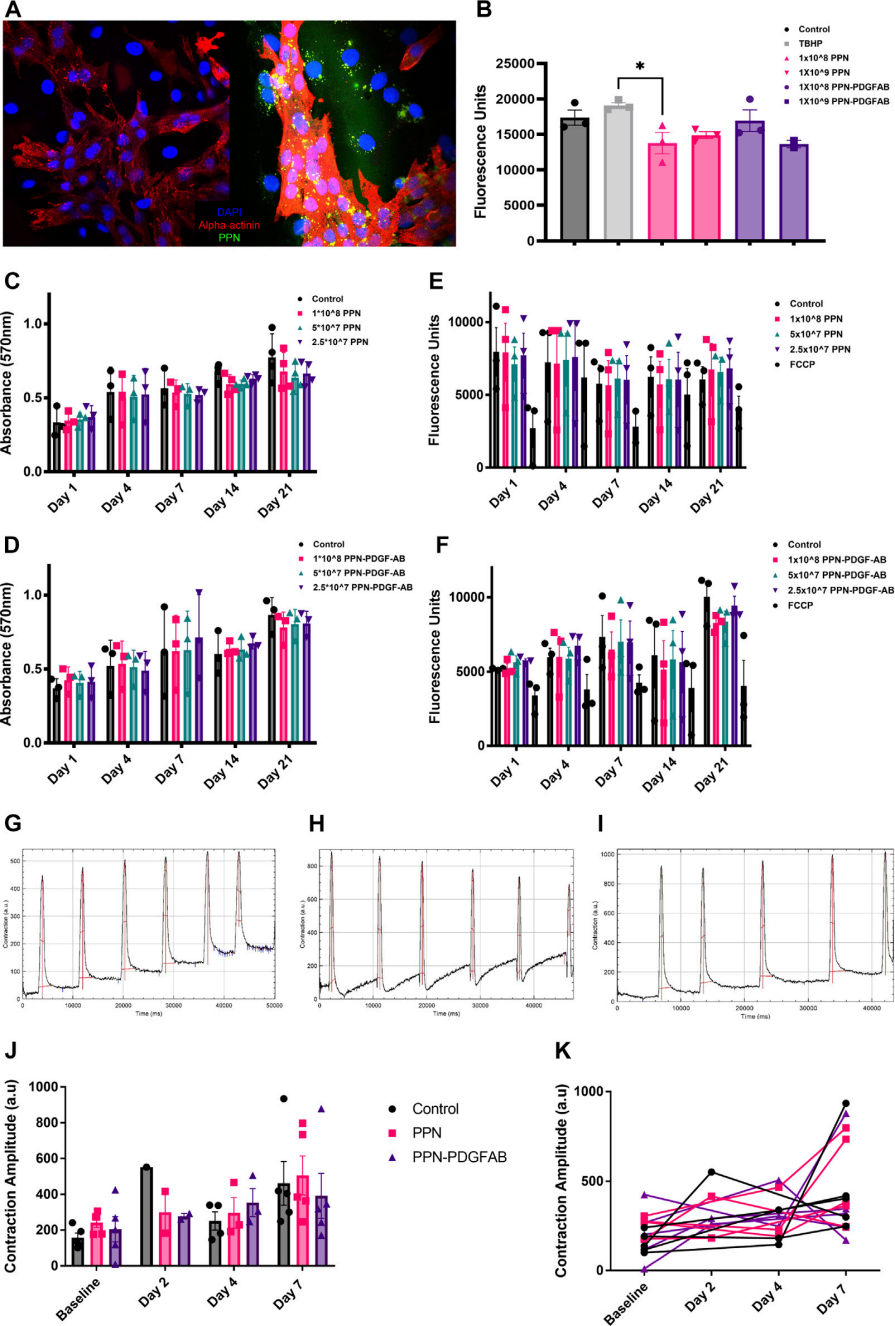
Intracellular presence of plasma polymerized nanoparticles is non-toxic to cardiomyocytes. **(A)**. Confocal images of NRVMs and NRVMs treated with PPN, showing intracellular PPN aggregates (green). **(B)**. Fluorescence intensity after 45 min incubation of DCFDA with pluripotent stem cell derived cardiomyocytes 24 h after treatment with PPN or PPN-PDGFAB. **(C, D)**. Optical density after 4h incubation of MTT with NRVMs 1-, 4-, 7-, 14- and 21-day after treatment with PPN **(C)** or PPN-PDGF-AB **(D)**. **(E, F)**. Mitochondrial membrane activity measured by TMRE fluorescence intensity in NRVMs 1-, 4-, 7, 14- and 21-day after treatment with PPN **(E)** or PPN-PDGF-AB **(F)**. **(G–I)**. Representative contraction amplitude traces from control **(G)** PPN treated **(H)** and PPN-PFGF-AB treated PSC-CMs, generated using the MUSCLEMOTION plugin for FIJI. **(J)**. Summary data showing mean contraction amplitude of PSC-CMs treated with PPN or PPN-PDGF at baseline and at 2-, 4- and 7-day post-treatment, in comparison to untreated controls. **(K)**. Individual well data showing contraction amplitude of PPN, and PPN-PDGF-AB, treated PSC-CMs over time, compared to untreated controls.

Plasma polymerized nanoparticles readily crossed the cardiomyocyte membrane and formed intracellular aggregates observable by microscopy (Figure 2A). We sought to determine whether the presence of intracellular PPN is detrimental to cardiovascular cells, particularly non-dividing cardiomyocytes. We investigated for potential cytotoxic effects of PPN or PPN-PDGF-AB in cardiomyocytes and coronary artery vascular smooth muscle cells, using physiologically relevant doses of PPN/PPN-PDGF-AB. The PPN-PDGF-AB doses were chosen based on the surface area of the nanoparticles and the size of free rhPDGF-AB to approximate a dose of 50 ng/mL and lower and higher dose equivalents.

To detect signs of cellular stress, we performed a ROS assay on iPSC-CMs 24 h after continuous incubation with PPN or PPN-PDGFAB. The fluorogenic dye, DCFDA, is taken up by cells and oxidized by hydroxyl, peroxyl, and other ROS to the highly fluorescent compound, 2′, 7′ –dichlorofluorescein (DCF). DCF fluorescence levels were not significantly different between control wells and those treated with nanoparticles, therefore we found no evidence of increased ROS production by PPN or PPN-PDGFAB treated cardiomyocytes at this timepoint, despite the presence of abundant intracellular PPN aggregates (Figure 2B). We did observe a significant reduction in DCF fluorescence in cardiomyocytes treated with 1 × 10^8^ PPN, compared to those treated with the TBHP positive control compound.

We used the MTT assay as a measure of cell viability. We found no effect of treatment with PPN or PPN-PDGFAB on NRVM or vascular smooth muscle cell viability at any of the doses or time-points tested (Figures 2C, D, Supplementary Figure S1). We then performed TMRE assays to detect changes in NVRM mitochondrial membrane potential in response to PPN or PPN-PDGF-AB. TMRE, a positively charged fluorescent dye, is sequestered by active mitochondria due to the relative negative charge of the mitochondrial matrix. Stressed or dying mitochondria experience a breakdown of their membrane potential and therefore do not accumulate TMRE. We found no significant reduction in TMRE fluorescence in NRVMs at 1, 7, 14, or 21 days after incubation with PPN and PPN-PDGF-AB, compared to controls. An inhibitor of mitochondrial oxidative phosphorylation, FCCP, was used as a positive control and reduced TMRE fluorescence measurements by approximately 50% (Figures 2E, F).

To determine whether the presence of intracellular PPN detrimentally impacts cardiomyocyte contractile function, we recorded beating pluripotent stem cell derived cardiomyocytes (PSC-CMs) and used a FIJI plugin, MUSCLEMOTION, to measure contraction amplitude before and up to 7 days after application of 1 × 10^9^ PPN or 1 × 10^9^ PPN-PDGF-AB or PBS control. We found a significant effect of time in culture on cardiomyocyte contraction amplitude, which increased in all treatment groups over the 7-day period. There was no difference in mean contraction amplitude between control wells and PPN or PPN-PDGF-AB treated wells at baseline or at 4 days or 7 days post-treatment (Figures 2G–J). As there is inherent variability in PSC-CM contraction amplitude between wells, plotting repeated measurements for individual wells confirmed that there is no decline in PPN treated cardiomyocytes’ contractile activity relative to their own baseline (Figure 2K).

### 3.3 PDGF-AB remains functional when bound to PPN

**FIGURE 3:**
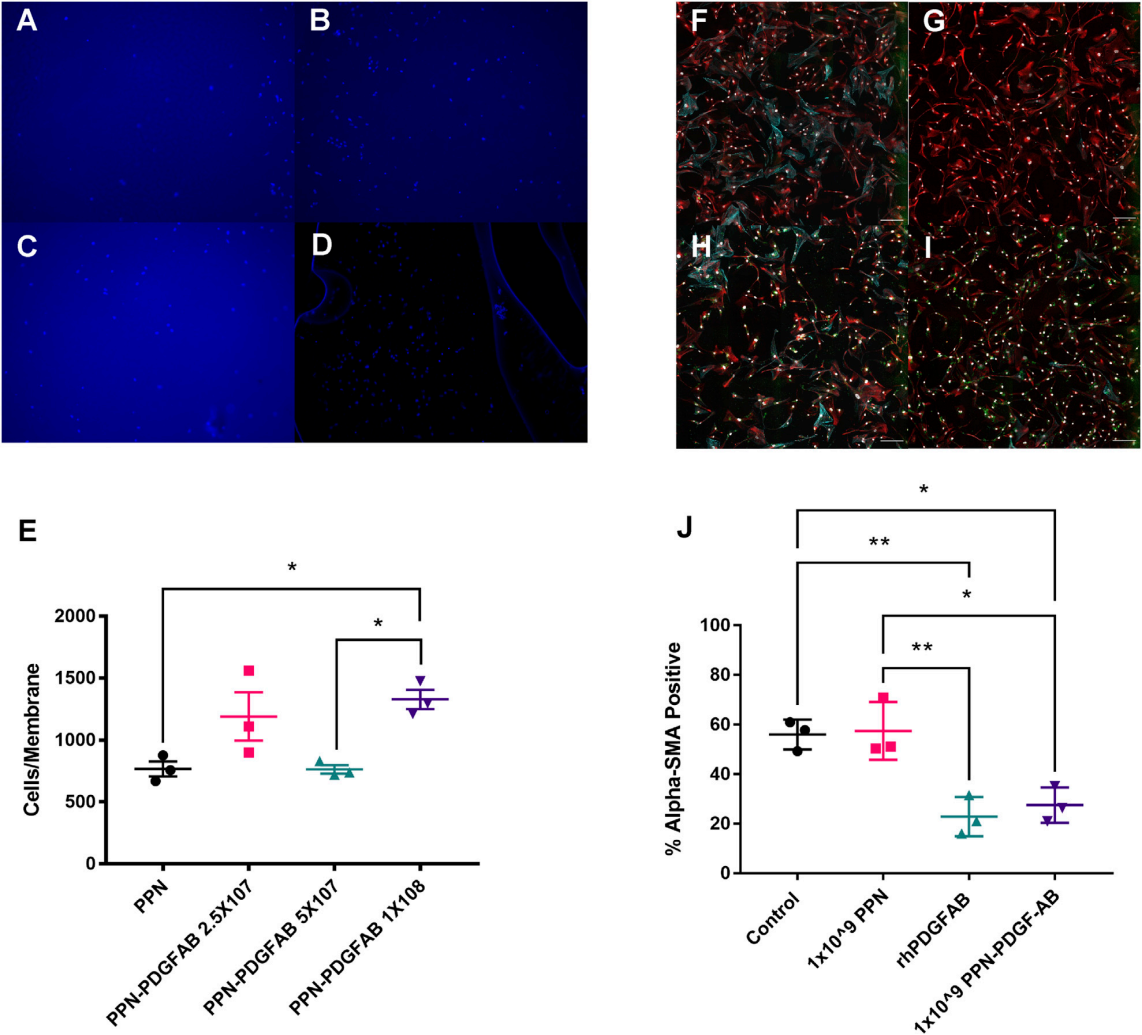
PDGF-AB remains functional when bound to plasma polymerized nanoparticles. **(A**–**D)**. Transwell migration assays. Images of DAPI stained nuclei of HCASMCs that migrated through to the underside of the Boyden chamber membrane in wells that contained 1 × 10^8^ PPN only **(A)** or 2.5 × 10^7^ **(B)**, 5 × 10^7^ **(C)**, or 1 × 10^8^ **(D)** PPN-PDGF-AB. Scale bars: 50 µm **(E)**. Quantitative analysis of transwell migration assays, showing increased migration of HCASMCs through membranes from wells containing 2.5 × 10^7^ and 1 × 10^8^ PPN-PDGF-AB, compared to PPN only. **(F–I)**. Expression of alpha smooth muscle actin (α-SMA) by human cardiac fibroblasts. Representative images of serum-starved cardiac fibroblasts **(F)** taken post-treatment with rhPDGFAB **(G)**, cargo-free PPN **(H)** and PPN-PDGFAB **(I)**. **(J)** Quantitative analysis shows PPN-PDGFAB reduces alpha-SMA expression by human cardiac fibroblasts to a similar extent as unbound rhPDGF-AB. Application of cargo-free PPN has no effect on α-SMA expression. Red-vimentin; teal—α-SMA; green—nanoparticle autofluorescence; white—DAPI. n = 3 biological replicates per group. Scale bars: 300 µm *p < 0.05, **p < 0.01.

We next sought to confirm that PDGF-AB protein remains functional when bound to PPN. PDGF is a known chemoattractant for PDGFRα+ human vascular smooth muscle cells, so we used a boyden chamber migration assay to measure the cells’ response to PPN-PDGF-AB (Figures 3A–D). A significantly increased number of cells migrated through the chamber membrane in wells containing media with PPN-PDGF-AB compared to those containing media with unbound PPN only (PPN 768 ± 60 vs. 1 × 10^8^ PPN-PDGF-AB 1329 ± 78 nuclei) (Figure 3E).

Following a tissue injury such as myocardial infarction, fibroblasts undergo activation to myofibroblasts and contribute to fibrosis via deposition of extracellular matrix components, such as collagens and elastin. The most commonly used marker of activated (myo)fibroblasts is alpha smooth muscle actin (Ivey and Tallquist, 2016). PDGF isoforms play a complex role in fibroblast activation and ECM accumulation, with PDGF-AB having previously been shown to reduce the expression of alpha smooth muscle actin in cardiac fibroblasts (Hume et al., 2023). We performed immunocytochemistry staining for αSMA and vimentin on human cardiac fibroblasts treated with PPN and PPN-PDGFAB (Figures 3F–I). We found that the percentage of alpha smooth muscle actin positive cardiac fibroblasts was significantly reduced in CFs treated with PPN-PDGF-AB and free PDGF-AB, compared to CFs treated with control vehicle or the cargo-free PPN (Serum starved (control) 56.0% ± 3.5% vs. PPN 57.5% ± 6.7% vs. rhPDGF-AB 22.9% ± 4.6% vs. PPN-PDGF-AB 27.5% ± 4.1%) (Figure 3J).

### 3.4 PPN-PDGF-AB in a rodent model of myocardial infarction

After confirming PDGF-AB remains functional while bound to PPN, we sought to determine whether PPN-PDGF-AB is cardioprotective in a rat model of myocardial infarction. Twenty-nine animals underwent surgery for permanent ligation of the left anterior descending artery (LAD) to induce myocardial infarction. All received PPN-PDGF-AB (1 × 10^9^ particles per dose) or equivalent doses of PPN, rhPDGF-AB, or saline control, injected into the infarct border regions immediately following reperfusion. Twenty-six animals recovered and reached the study endpoint at 14 days post-infarct. Three animals succumbed to large infarcts during surgery or during the 24-h period immediately following surgery.

**FIGURE 4:**
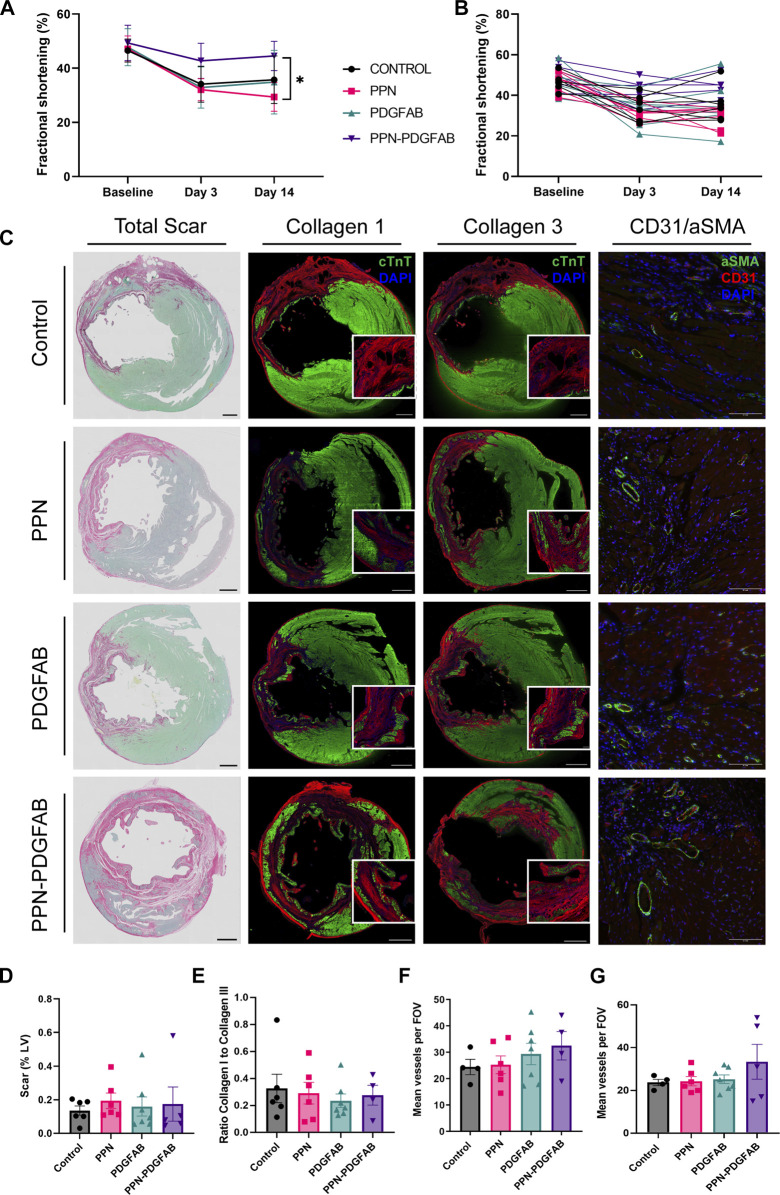
PPN-PDGFAB delivery after myocardial infarction preserves fractional shortening. **(A)**. Fractional shortening summary data and individual animal data **(B)** at each time point. Sample sizes were n = 5 (PPN-PDGFAB), n = 6 (Control), and n = 7 (PPN and PDGFAB) biological replicates. **(C)**. Representative images of total apical infarct scar, infarct collagen I, infarct collagen III, and CD31+/αSMA + vessels in the infarct border zone. Scale bars: 1 mm. Inset scale bars: 200μm **(D)**. Quantitative analysis of picrosirius red fast green stained sections, showing infarct scar size as a percentage of LV area at the apex. **(E)**. Ratio of infarct collagen I to collagen III at the apical level. **(F, G)**. CD31+/αSMA + vessel density at the apical infarct core and infarct border zone.

To measure LV function, we performed transthoracic echocardiography at baseline, and at 3 days and 14 days post-infarction. At 3 days post-infarct, infarct burden was comparable between all treatment groups, as measured by fractional shortening. At 14- days post-infarct, rats treated with PPN-PDGF-AB had significantly improved fractional shortening compared to rats treated with PPN only (day 14 44.5% ± 2.4% for PPN-PDGF-AB vs. 29.4% ± 2.0% for PPN), attributable to preserved systolic function as measured by LVESD (Figures 4A,B, Supplementary Figure S2, Supplementary Table S1). Echocardiography analysis was performed by 2 operators, with good inter-operator agreement (Supplementary Figure S3). Two animals were excluded from analyses due to failure of infarct creation as determined by the absence of reduction in fractional shortening at day 3 and total absence of infarct scar at the apical and mid LV levels as measured by histology.

To investigate possible underlying mechanisms for the preservation of fractional shortening in PPN-PDGFAB treated hearts, we collected tissue for histology and immunohistochemistry analyses at day 14 post-infarct. To determine if PPN-PDGF-AB treatment affected infarct scar size, we quantified scar as a percentage of the total LV area, using picrosirius red and fast green staining at the apex and mid-ventricle levels. We found that infarct scar sizes were not significantly different between groups at either level (Figures 4C, D, Supplementary Figures S4A–D, S5). Overall scar burden was higher at the apex, so we first focused on measuring scar composition and vessel density at this level.

We performed immunohistochemistry targeting collagen I and collagen III in adjacent tissue sections. We then quantified the ratio of collagen I to collagen III. At this timepoint (day 14 post-infarct) collagen III was the predominant collagen subtype present in all scars, however we found no differences in the ratio of type I to type III between the treatment groups (Figure 4E).

As PDGF-AB is known to be angiogenic during the proliferative stage of wound healing, we hypothesized that enhanced growth of new blood vessels may have salvaged at-risk myocardium in the infarct border regions. At the apical level, there were no significant differences in infarct core region or border zone vessel density (Figures 4F, G). We did observe a trend towards increased vessel density in the mid-LV border zone of PPN-PDGF-AB treated infarcts, compared to controls and a significantly increased vessel density in the mid-LV infarct core region of PDGFAB treated hearts compared to both Control and PPN-PDGFAB treated hearts (Border zone 50 ± 7 vessels per FOV for PPN-PDGF-AB vs.30 ± 4 vessels for Control, *p* = 0.0797; Infarct core 52 ± 7 vessels per FOV for PDGFAB vs. 24 ± 5 vessels for Control (*p* = 0.0137) and 30 ± 3 vessels for PPN-PDGFAB (*p* = 0.0491), Supplementary Figure S5).

## 4 Discussion

Preclinical evidence suggests PDGF-AB has therapeutic potential for repair and functional recovery of the acutely infarcted heart (Asli et al., 2019; Thavapalachandran et al., 2020). There is precedent for the use of PDGF clinically, with PDGF-BB products already available for the treatment of chronic wounds and periodontal defects (Smiell et al., 1999; Nevins et al., 2005). However, the pro-angiogenic and pro-proliferative effects of PDGF receptor signaling have been implicated in the progression of cancers and the development of atherosclerosis, organ fibrosis, and pulmonary hypertension (Bonner, 2004; Raines, 2004; Barst, 2005; Farooqi and Siddik, 2015). The potential for unwanted off-target effects of continuous systemic delivery of PDGF-AB combined with the prospect of increasing availability of PDGF-AB to the infarct and border zone region prompted us to investigate a nanocarrier-based method for localised delivery to cardiac cells.

Plasma polymerized nanoparticles (PPN) are a new class of nanocarriers which allow for a facile, one-step functionalization with molecular cargo. In contrast with conventional wet chemistry approaches, PPN are produced in a dry plasma medium via plasma polymerization (Santos et al., 2016) of a reactive gaseous mixture comprising of nitrogen, argon and acetylene (Santos et al., 2018). Activation of acetylene-containing mixtures in a rf discharge generates complex dusty plasmas, which are populated with high concentrations of electrostatically stabilized nanoparticles. Control over the formation, yield and physical-chemical properties of PPN (e.g., size, surface chemistry and charge) is achieved by selection of relevant input parameters, including system pressure, gas flow rates, pumping efficiency and input rf power. The extraction of PPN from the plasma medium is carried through exposure of a well-shape substrate (polystyrene well plates) to the dusty plasma, enabling high yield collection and further storage of PPN in their native dry state up until before the conjugation process with molecular cargo in solution (Santos et al., 2019).

Here, we selected plasma input parameters that yield pristine PPNs with a surface chemistry characterized by the presence of functional groups (e.g., amines and carboxylic acid). Dispersion of PPN in aqueous solution yields aggregation-free nano-formulations, stabilized by electrostatic repulsion due to protonation of amine functional groups. Immobilization of PDGF-AB to PPN was performed in a one-step mixing and incubation process in water. The stability of the resulting PPN-PDGF-AB formulations was demonstrated by unchanged hydrodynamic size and PDI compared to pristine PPN. The decrease in zeta potential of PPN-PDGF-AB relative to unfunctionalized PPN suggests changes to the ionic double layer upon binding, hence confirming immobilization and retention of PDGF-AB on the surface of PPN. The resulting net charge of the nano-constructs stabilized dispersion in solution, resulting in a monodisperse nano-population without aggregation.

PPN readily crossed the cell membrane and intracellular aggregates were still visible 3 weeks after delivery. Although PPN have been previously proven non-cytotoxic in multiple cell lines, cardiomyocytes present a unique environment due to their complex contractile machinery and their inability to dilute PPN aggregate build up through proliferation. Despite these additional considerations, we did not find evidence of cellular stress or cytotoxicity in PPN, or PPN-PDGF-AB treated cardiomyocytes. The MTT assay is a non-specific measure of viability and can be influenced by cell number, proliferation rate, MTT concentration, and incubation time (Ghasemi et al., 2021). We controlled for all factors except inherent variability in proliferation rate of non-myocytes and between HCASMCs from different donors. Nevertheless, we observed that biological replicates behaved similarly in their response to PPN and PPN-PDGF-AB.

We also measured cardiomyocyte mitochondrial membrane potential and contractile activity. It is common to see variability between PSC-CM differentiations and within PSC-CM differentiations recorded on different days. Unexpectedly, we observed reduced contractile amplitude at 7 days post-treatment in some wells treated with PPN-PDGF-AB, but not with PPN only. This suggests any inhibitory effect may be mediated via PDGF-AB rather than the nanoparticles themselves. Potentially, there is a stimulatory effect of PDGF-AB on stem cell derived PDGFRα+ non-myocytes present in the well, which influences the contractile properties of surrounding PSC-CMs (Huang et al., 2020).

The PDGF-A and PDGF-B chains contain highly conserved regions known to be crucial for receptor binding (Clements et al., 1991; Ostman et al., 1991; Fenstermaker et al., 1993). For the PDGF-AB heterodimer to remain functional while bound to PPN, these monomer binding sites must remain available to PDGF receptors. We confirmed that human PDGFRα+ vascular smooth muscle cells and cardiac fibroblasts respond to treatment with PPN-PDGFAB in the same manner as they do to free recombinant human PDGF-AB. We saw increased migration of coronary artery vascular smooth muscle cells in response to PPN-PDGF-AB, as well as a striking reduction in alpha smooth muscle actin expression in cardiac fibroblasts treated with recombinant human PDGF-AB protein and with PPN-PDGF-AB.

We observed a modest preservation of fractional shortening at day 14 in animals treated with PPN-PDGF-AB after permanent LAD ligation, compared to those treated with PPN only. However, this was not accompanied by significant changes in scar composition or peri-infarct vascularity. A single dose of PDGF-AB or PPN-PDGFAB direct to the myocardium post-infarct does not appear to be sufficient to induce cardiac repair. Given that pretreatment with PPN-PDGF-AB is the less clinically applicable scenario, an increased or repeat dosage of PPN-PDGF-AB post infarct may be required for robust efficacy of PDGF-AB in this model.

One limitation of this study is that the PPN-PDGFAB treatment was given immediately following infarct by permanent ligation, so we were not able to collect post-infarct baseline echocardiography measurements of LVEDV and LVESV. A second limitation relates to the difficulty in accurately determining equivalent doses of recombinant PDGF-AB protein and PPN-PDGF-AB. We calculated the surface area of the nanoparticles and the size of the PDGF-AB protein to estimate total PDGF-AB bound to PPN. It is also not possible to compare this direct intramyocardial dose to the dose received by the heart when PDGF-AB was delivered systemically in earlier studies (Asli et al., 2019; Thavapalachandran et al., 2020).

Had we observed a beneficial effect of PPN-PDGFAB over PDGFAB our next steps would be to investigate potential mechanisms, including increased PDGF-AB stability, bioavailability, or other paracrine effects of PPN-PDGFAB. As it stands, this work establishes the possibility of using PPN as a platform for delivery of bioactive compounds directly to the heart. From a translational perspective, minimally invasive PPN delivery methods such as catheter-based intracoronary delivery or endocardial injections will be superior to direct intramyocardial injections. Larger, longer-term studies to compare these methods will require the use of clinically relevant pig or non-human primate models and should evaluate PPN biodistribution and bioavailability to the heart. This is particularly pertinent for any systemic intravenous or even intracoronary delivery method given that PPN are readily taken up by other organs (Michael et al., 2021).

In summary, we have performed simple conjugation of PDGF-AB to plasma polymerised nanoparticles for direct administration of functional protein to cardiac cells *in vitro* and *in vivo*. Our results demonstrate safety and feasibility of the PPN platform for delivery of therapeutics directly to the myocardium for either cell surface or intracellular interactions. Future work will focus on optimizing PPN-PDGF-AB formulations and determining the most effective dosage and timing to deliver PDGF-AB systemically, ultimately to improve bioavailability and efficacy of PDGF-AB and accelerate recovery of patients with heart failure following myocardial infarction.

## Figures and Tables

**Table:** 

**αSMA**	alpha smooth muscle actin
**CF**	cardiac fibroblast
**DCFDA**	2′,7′–dichlorofluorescin diacetate
**DLS**	dynamic light scattering
**DMEM**	Dulbecco’s modified eagle medium
**ECM**	extracellular matrix
**FFCP**	carbonyl cyanide 4-(trifluoromethoxy) phenylhydrazone
**FOV**	field of view
**HCASMC**	human coronary artery smooth muscle cell
**LAD**	left anterior descending (artery)
**LV**	left ventricle
**LVESD**	left ventricular end systolic diameter
**LVEDD**	left ventricular end diastolic diameter
**MEMα**	modified minimum essential medium
**MI**	myocardial infarction
**MTT**	3-(4,5-dimethylthiazol-2-yl)-2,5-diphenyltetrazolium bromide
**NFW**	nuclease free water
**NGS**	normal goat serum
**NRVM**	neonatal rat ventricular myocyte
**OES**	optical emission spectroscopy
**PDGF-AB**	platelet derived growth factor AB
**PDGFR**	platelet derived growth factor receptor
**PDI**	polydisperse index
**PPN**	plasma polymerized nanoparticles
**pSAX**	parasternal short axis
**PSC-CM**	pluripotent stem cell derived cardiomyocyte
**RF**	radiofrequency
**rhPDGF-AB**	recombinant human platelet derived growth factor AB
**ROS**	reactive oxygen species
**RPMI**	roswell park memorial institute
**SCVI**	Stanford cardiovascular institute
**TBHP**	Tert-butyl hydroperoxide
**TMRE**	Tetramethylrhodamine, ethyl ester
**UV/VIS**	ultraviolet-visible spectroscopy

## Data Availability

The raw data supporting the conclusion of this article will be made available by the authors, without undue reservation.
