# Supplementary material for: Plasma polymerized nanoparticles are a safe platform for direct delivery of growth factor therapy to the injured heart
Source: Front Bioeng Biotechnol. 2023 Jun 20;11:1127996. doi: 10.3389/fbioe.2023.1127996 (PMC10319252; doi:10.3389/fbioe.2023.1127996)
Supplement: Supplementary file 1 [file DataSheet1.zip › Data Sheet 1 (79).pdf]

Supplementary Methods

MTT viability assay on Human Coronary Artery Vascular Smooth Muscle Cells

HCASMCs were seeded on 48 well plates at $1.5x10^4$ cells per well. MTT assays were performed on days 1, 4 and 7 post-seeding, using the protocol and nanoparticle doses described previously for NRVMs.

PDGFRα expression on Human Coronary Artery Vascular Smooth Muscle Cells

HCASMCs were dissociated with TryPLE, washed, and stained with Fixable Viability Stain 585 (BD Biosciences; 1μL/mL). After subsequent wash steps, the cells were stained with mouse anti-human PDGFRα-BV786 (BD Biosciences; 1:20) and run on a BD LSRFortessa (BD Biosciences). Unstained, FVS575 only and PDGFRα only FMO controls were used. Analysis was performed using FlowJo v6.10 (BD).

Supplementary Figure Legends

Supplementary Figure 1. PPN and PPN-PDGFAB are non-toxic to PDGFRα positive human coronary artery vascular smooth muscle cells. a. Flow cytometry analysis confirming PDGFRα expression on primary human coronary artery vascular smooth muscle cells. b-c. Optical density after 4h incubation of MTT with human coronary artery vascular smooth muscle cells treated with PPN only (b) or PPN-PDGF-AB (c).

Supplementary Figure 2. Echocardiography measurements of left ventricular end systolic (LVESD) and end diastolic (LVEDD) diameters. a. Representative day-3 and day-14 M-mode echocardiography views taken from animals treated with saline control, PPN only, rhPDGF-AB, and PPN-PDGFAB.

Supplementary Figure 3. Echocardiography inter-operator variability. a-c. Bland-Altman plots showing variability between echocardiography operator fractional shortening measurements at pre-infarct baseline (a), Day 3 (b), and Day 14 (c) after permanent occlusion of the LAD. d. Inter-operator variability of all fractional shortening measurements depicted by Pearson's correlation plot.

Supplementary Figures 4a-d. Picrosirius red and fast green staining for left ventricular scar size analysis. a. Apex and mid LV sections from vehicle control treated hearts. b. Apex

and mid LV sections from PPN treated hearts. c. Apex and mid LV sections from PDGF-AB treated hearts. d. Apex and mid LV sections from PPN-PDGF-AB treated hearts. Scale bars: 1mm.

Supplementary Figure 5. Quantitative analysis of mid-LV infarct scar size and vessel density. a. Quantitative analysis of picrosirius red fast green stained sections, showing infarct size as a percentage of total LV area at the mid ventricle level. b-c. CD31+/αSMA+ vessel density at the mid-LV infarct core and infarct border zone.
