# Supplementary figures and images for: Plasma polymerized nanoparticles are a safe platform for direct delivery of growth factor therapy to the injured heart
Source: Front Bioeng Biotechnol. 2023 Jun 20;11:1127996. doi: 10.3389/fbioe.2023.1127996 (PMC10319252; doi:10.3389/fbioe.2023.1127996)

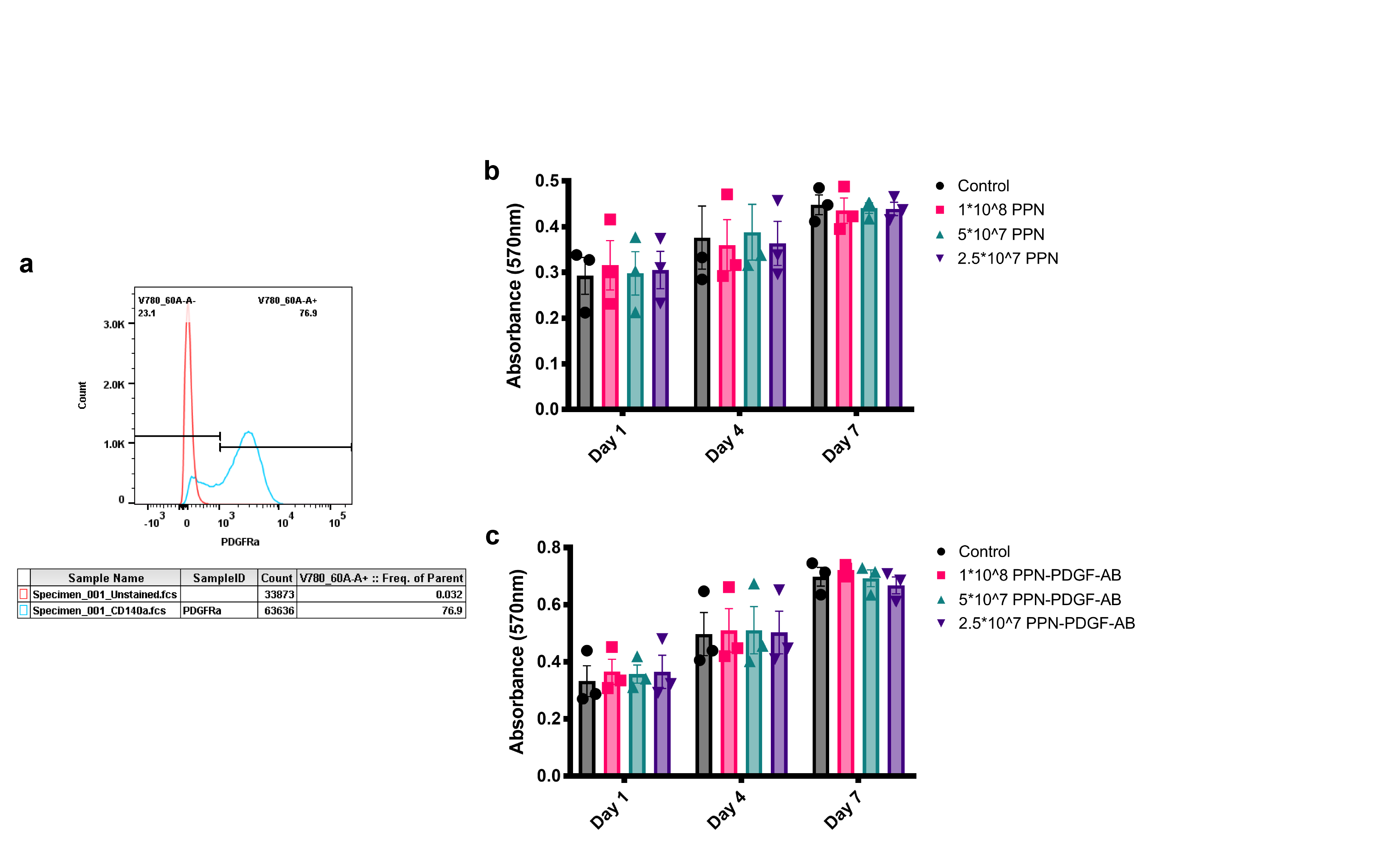

Supplement: Supplementary file 1 [file DataSheet1.zip › Supplementary Figure1 HCASMCs.tif]

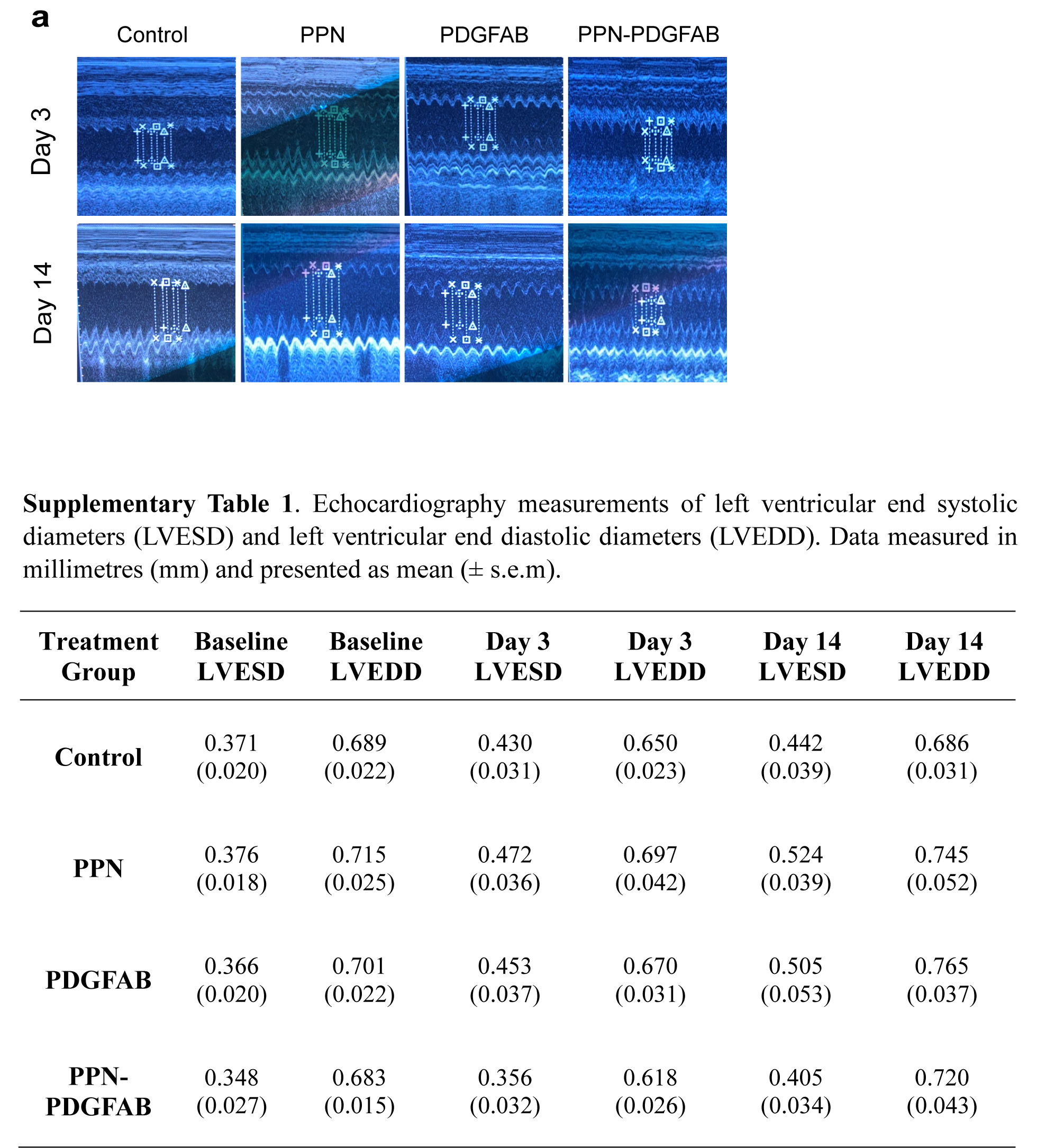

Supplement: Supplementary file 1 [file DataSheet1.zip › Supplementary Figure 2_LVEDS and LVEDD.tif]

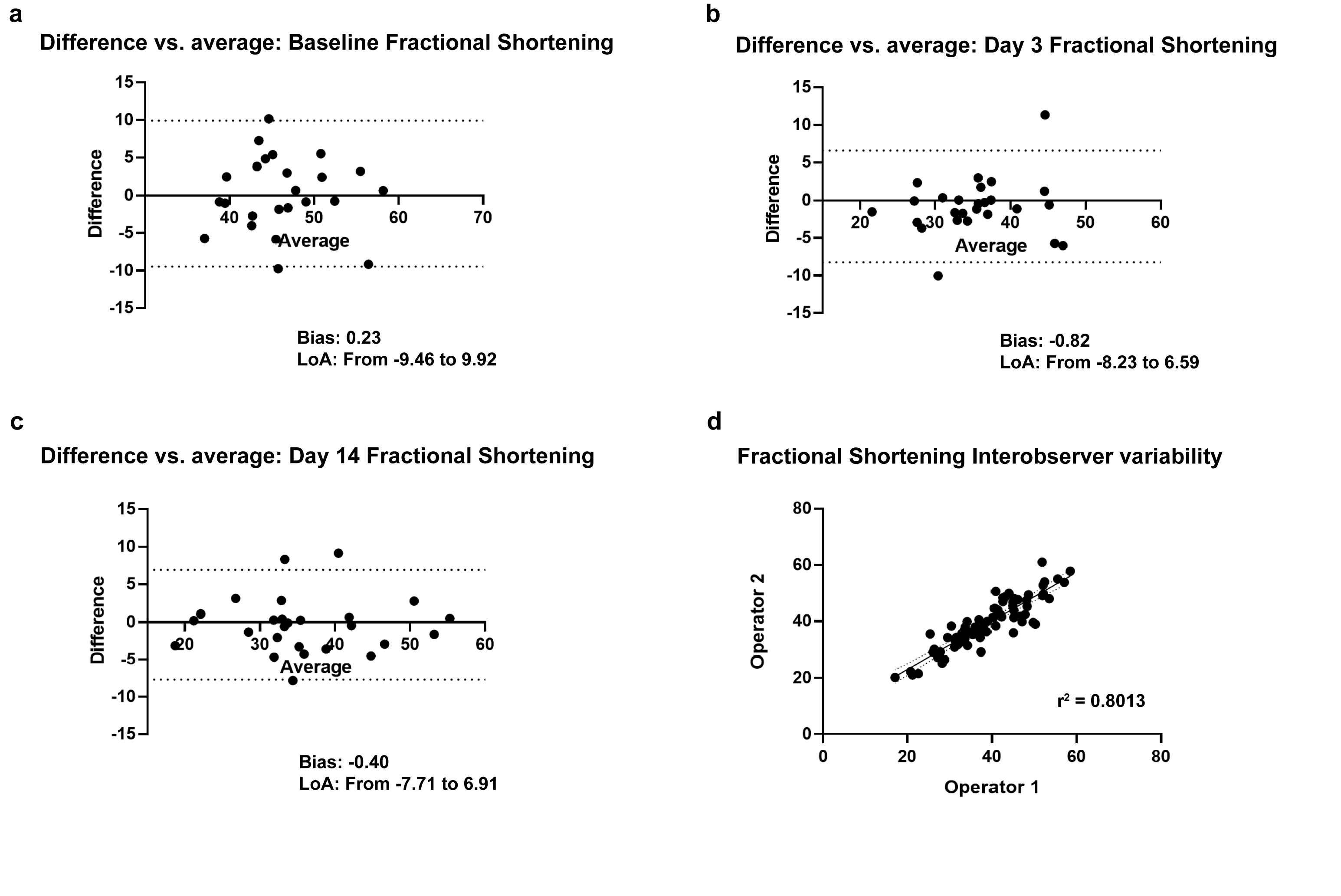

Supplement: Supplementary file 1 [file DataSheet1.zip › Supplementary figure 3_echocardiography interoperator variability.tif]

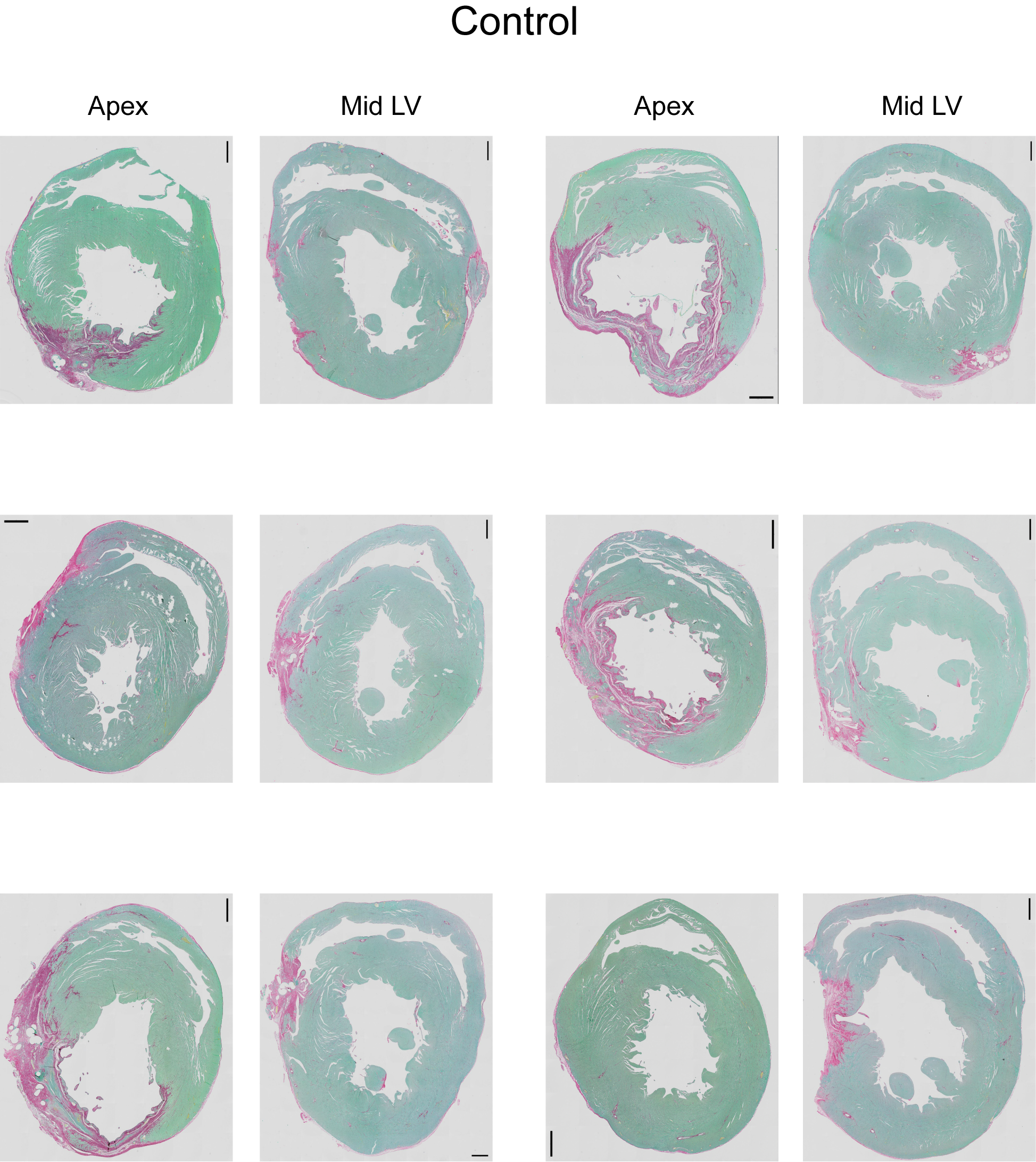

Supplement: Supplementary file 1 [file DataSheet1.zip › Supplementary figure_PSR sections vehicle treated.tif]

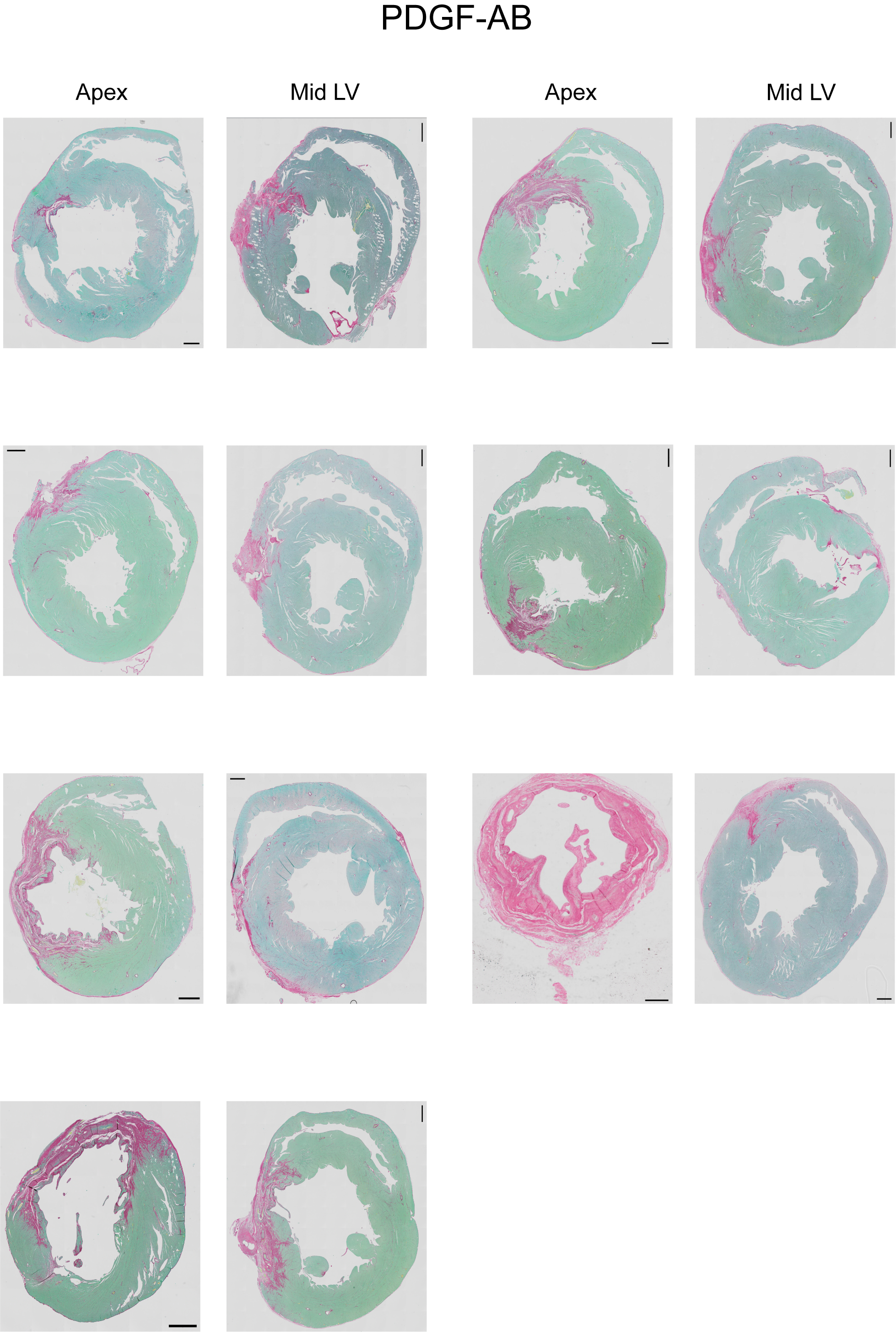

Supplement: Supplementary file 1 [file DataSheet1.zip › Supplementary figure_PSR sections PDGFAB treated.tif]

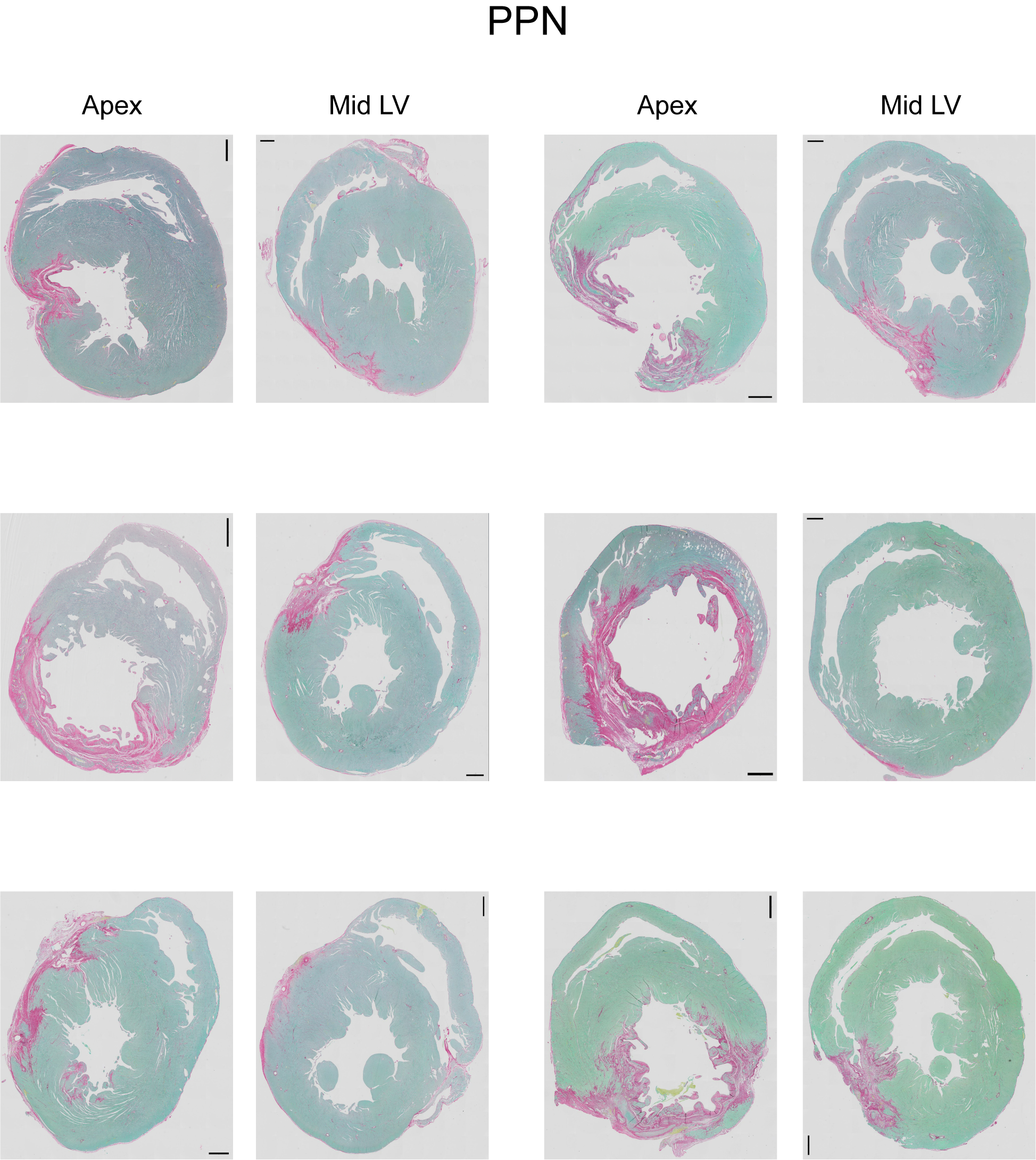

Supplement: Supplementary file 1 [file DataSheet1.zip › Supplementary figure_PSR sections PPN treated.tif]

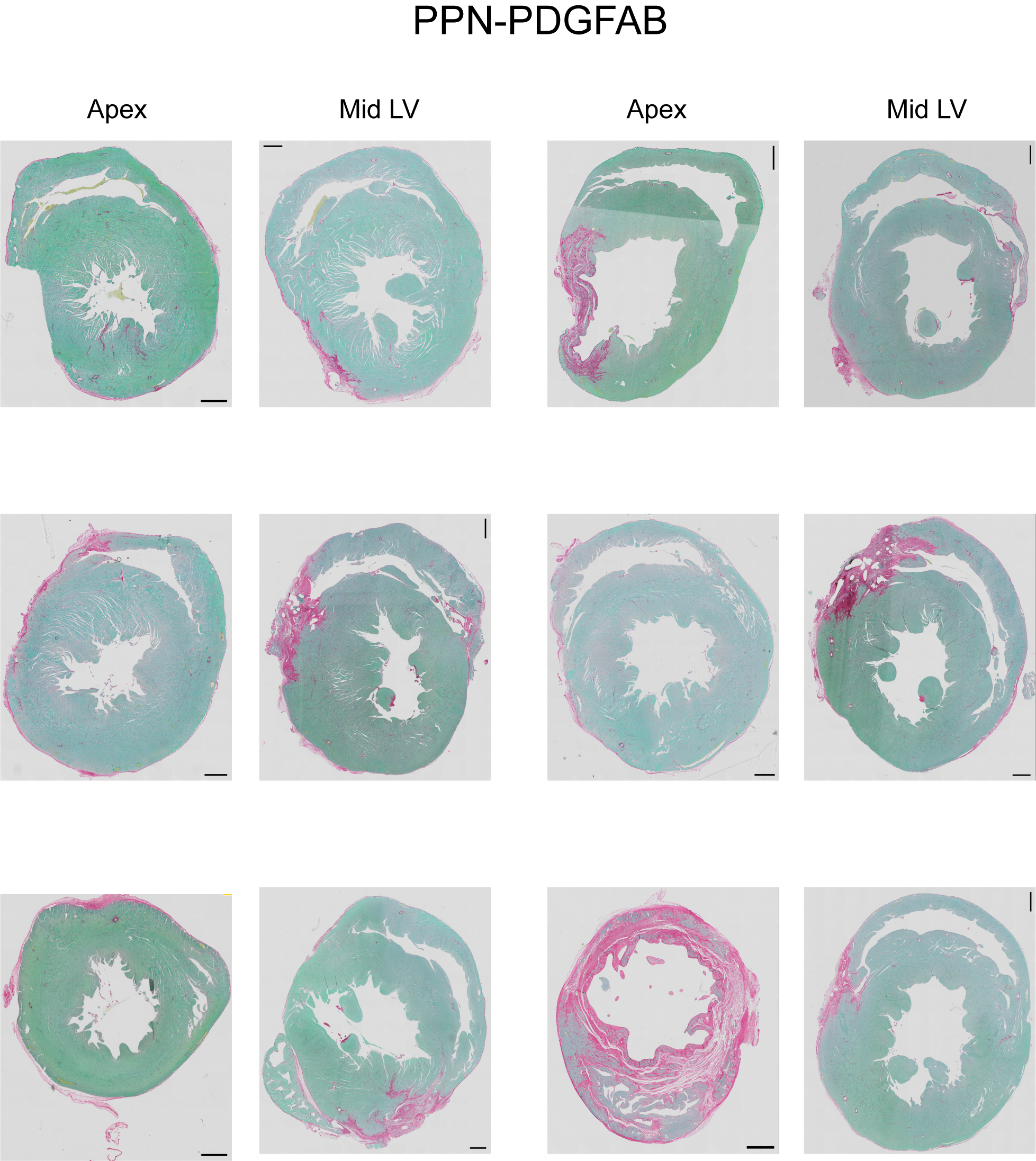

Supplement: Supplementary file 1 [file DataSheet1.zip › Supplementary figure_PSR sections PPNPDGFAB treated.tif]

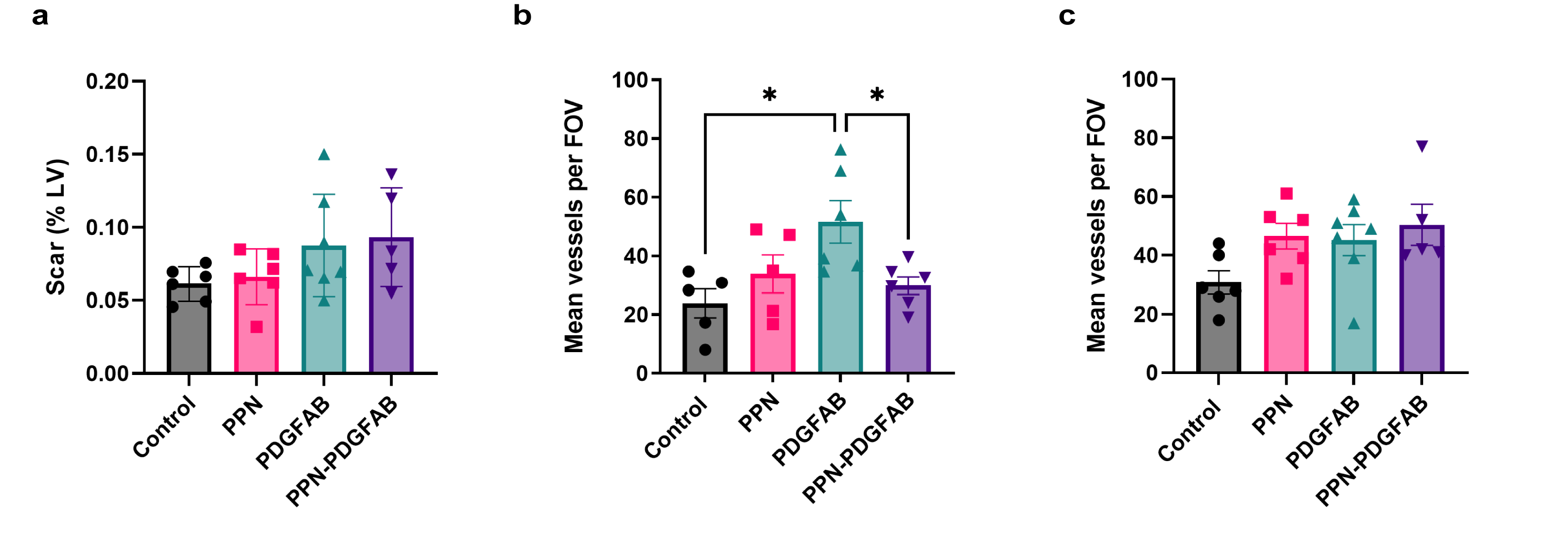

Supplement: Supplementary file 1 [file DataSheet1.zip › Supplementary Figure 5 Mid LV scar size and vessel density.tif]
